# The Use of Virtual Reality to Facilitate Mindfulness Skills Training in Dialectical Behavioral Therapy for Spinal Cord Injury: A Case Study

**DOI:** 10.3389/fpsyg.2018.00531

**Published:** 2018-04-23

**Authors:** Araceli Flores, Marsha M. Linehan, S. Rob Todd, Hunter G. Hoffman

**Affiliations:** ^1^Ben Taub Hospital Psychiatry and Behavioral Sciences, Baylor College of Medicine, Houston, TX, United States; ^2^Behavioral Research and Therapy Clinics, Psychology, University of Washington, Seattle, WA, United States; ^3^General Surgery at Ben Taub Hospital, Surgical Intensive Care Unit at Baylor St. Luke's Medical Center, Baylor College of Medicine, Houston, TX, United States; ^4^VR Research Center at Human Photonics Lab, Mechanical Engineering, University of Washington, Seattle, WA, United States

**Keywords:** virtual reality therapy, dialectical behavior therapy, mindfulness training, spinal cord injury (SCI), emotions

## Abstract

**Introduction:** Paralysis from a spinal cord injury (SCI) increases risk of psychological problems including suicide attempts, substance use disorder, negative emotions (e.g., anger), depression, anxiety, ASD/PTSD. Dialectical Behavioral Therapy® (DBT®) has been shown to be effective for treating similar psychological symptoms in non-SCI patient populations. The current study explored for the first time, the feasibility and clinical potential of using Immersive Virtual Reality (VR) enhanced DBT® Mindfulness skills training to help reduce psychological symptoms (negative emotions and anxiety, ASD/PTSD) of two patients with SCI. Patient 1 was a 39-year-old male patient suffering multiple spinal cord injuries, resulting in quadriplegia, after falling out of a four story building. Patient 1 had severe depression, and anxiety symptoms. Patient 2, was a 31 year old male with a C7 vertebral body fracture, leading to paresis, after suffering a blunt force trauma injury during an attempted suicide, jumping from a moving vehicle. Patient 2 had mild depression, and anxiety symptoms.

**Methods:** Each patient looked into VR goggles, and had the illusion of slowly “floating down” a river in virtual reality while listening to DBT® Mindfulness Skills training instructions. Each patient filled out brief psychological ratings before and after each VR session, four VR DBT® sessions for patient 1, and two VR DBT® sessions for patient 2.

**Results:** As predicted, patient 1 reported reductions in negative emotions after each VR DBT® Mindfulness session. Patient 2 had mixed results on some of the measures of negative emotions. And both patients reported feeling less depressed, less anxious, and less emotionally upset, after VR DBT® Mindfulness Skills learning. Patient 2 reported large reductions in short term ASD/PTSD symptoms after his first VR DBT® mindfulness skills training session.

**Conclusion:** This study explored the feasibility of using VR DBT® with quadriplegic or paresis SCI patients. Both SCI patients accepted VR, the patients liked using VR, and, with assistance from the therapist, the patients were able to use the VR equipment, despite being paralyzed. Additional research and development will be needed to determine whether VR DBT® Mindfulness Skills training leads to any long term improvements in outcome.

## Introduction

Approximately 15,000 new cases of traumatic spinal cord injury (SCI) occur each year, in the United States alone (National Spinal Cord Injury Statistical Center, 2018). The most common causes of spinal injuries are motor vehicle accidents, violence (e.g., getting shot in the neck or getting shot in the back), falls, suicide attempts, and recreational activities. The spinal cord is the cable of nerves used by the human brain to send signals to and from different parts of the body. If there is damage to the spinal cord, the person's limbs can become partially or completely paralyzed. If a patient with complete paralysis wants to move their arm, their brain tries to send signals to the arm, but now the signal from the brain only travels part way down the spinal cord, and the signal stops where the spinal cord is injured (the lesion). Usually the closer to the brain the spine is damaged, the more limbs paralyzed. Some patients initially show paralysis, but regain some functionality and sensation, over time (e.g., when the swelling goes down in their spine). For example, patients who still have some signals passing through the spinal lesion can benefit from rehabilitation exercises, as one way to reduce paralysis/increase functionality.

In addition to extensive medical treatments of their physical injuries, and physical rehabilitation, patients with severe spinal cord injuries must often go through difficult psychological adjustments, beginning in the acute phase of their recovery (e.g., during early hospitalization within 2 weeks after suffering a blunt force trauma injury). Patients who have recently become paralyzed are often anxious about their future, wondering how long they are going to live, and worrying about what they will do now that they are paralyzed. SCI patients often feel helpless, and report poor quality of life. Social support increases resiliency. After injury, patients with spinal cord injuries often cannot return to the same job or social activities they had before injury, and they typically do not socialize as much, reducing their social support. Their circle of friends, significant others, family, and co-workers typically gets smaller, after someone becomes paralyzed. Spinal injury patients often have psychological problems such as low total self-concept, acute stress disorder/PostTraumatic Stress Disorder (PTSD) symptoms, and/or suicidal thoughts and behavior (Jurisić and Marusic, 2009; Kennedy and Garmon-Jones, 2017). According to a review by Pollock et al. (2017), patients having negative thoughts and emotions related to their injury have lower levels of acceptance of their injury, poorer adjustment to being disabled, and report more severe PTSD symptoms (Pollock et al., 2017).

It is important for spinal injury patients to learn effective coping skills to help them deal with difficult changes in lifestyle associated with having an abrupt, severe, and often permanent loss of mobility. Clinical psychologists can help spinal injury patients adjust to psychological issues and patients can learn techniques to help them reduce negative emotions. However, some patients never receive psychological treatment and SCI patients usually do not receive psychological treatments during the acute phase of their recovery, during the first 2 weeks of hospitalization after a traumatic spine injury, because during the acute phase, patients are typically in survival mode, medically. A new “low risk, low stress, simple to administer” psychological intervention that could help patients avoid getting too negative during the acute phase of their physical recovery, would be welcomed and has the potential to help prevent development of severe long term psychological problems.

Mindfulness is a simple psychological technique that helps people learn how to “be in the present moment, without judgement” and helps patients gain more control over their own pathological thought patterns (e.g., to become less critical of themselves and others). Dialectical Behavioral Therapy (DBT®) is effective for treating a number of psychological problems, such as suicidal thoughts, depression, negative emotion (anger), and anxiety, (e.g., Linehan, 1993, 2015; Soler et al., 2012; Linehan et al., 2015; McClure et al., 2015; Valentine et al., 2015; Elices et al., 2017; Kramer, 2017). Many psychological problems commonly suffered by SCI patients could potentially benefit from DBT®, however, to date, there are no PubMed indexed studies exploring the use of DBT® or DBT® skills learning with spinal injury patients.

Researchers have recently begun exploring the feasibility of using virtual reality technology to help patients learn how to practice mindfulness. Virtual reality is a very attention grabbing technology that helps patients focus their attention on achieving a state of mindfulness. In exploratory case studies, Virtual Reality DBT® mindfulness skills learning has recently been shown to reduce negative emotions in a patient with borderline personality disorder (Navarro-Haro et al., 2016) and in a severe burn injury patient (Gomez et al., 2017). Immersive virtual reality occludes patients view of the real world (Hoffman, 2004), which may help beginner patients achieve a state of mindfulness more easily. In addition, VR may make mindfulness more interesting, increasing compliance with mindfulness homework exercises.

The current case study explores the feasibility of using Virtual Reality Dialectical Behavioral Therapy Mindfulness Skills Training for two patients in acute hospital care for recent spinal cord injuries. SCI patient 1 (quadriplegic) was reporting strong negative emotions associated with their recent SCI after falling from a four story building, in an apparent suicide attempt. Patient 1 was assessed to have depression and anxiety. SCI patient 2, (paresis) had mild depression on the brief medical BDI, and reported some evidence of anxiety on the STAI-Y.

As a first step toward eventually using full standard DBT® (Linehan, 2015) with SCI patients, the current study is the first PubMed published study to explore the feasibility and acceptability of using immersive Virtual Reality DBT® mindfulness skill training with SCI patients. We predicted that despite their full or partial paralysis, the SCI patients would be able to use the VR equipment, they would like VR DBT®, and they would want to use VR DBT® again. We further predicted that after VR DBT®, the two spinal injury patients would report fewer negative emotions and less anxiety, and/or fewer acute stress disorder/PTSD symptoms. Both patients were in the hospital recovering from recent traumatic spinal cord injuries (2 weeks earlier).

## Materials and methods

This study was approved by the Ben Taub Hospital IRB, and each participant signed a consent form, and gave written permission to publish their de-identified results. The participants were not compensated for their participation in this study. Patient 1 was a 39-year-old male patient hospitalized with a traumatic SCI C4-5 resulting in quadriplegia and respiratory failure requiring trachiotomy (breathing through a hole in his throat) after falling out of 4 story building (apparent suicide attempt) 2 weeks prior to the current study. The fall/accident resulted in C6/C7 Burst Fracture, C4/C5 Spinous Process Fractures, C5/C6 Transverse Process Fractures, SCI, and right 8th through 11th rib fractures. Psychological assessments determined that the patient was clinically depressed, and had high anxiety associated with the traumatic event that left him paralyzed with a SCI. He could not remember anything about events leading up to his fall out of a building and he did not have any PTSD symptoms.

Patient 2 suffered a SCI after jumping out of a moving vehicle, in an attempted suicide. Due to damage to the spine including a C7 fracture, the hospitalized patient had bilateral hand numbness and parasthesias, and paresis (partial paralysis of his legs). Both patients were treated by the first author of this paper (AF). The psychotherapist (AF) has a Ph.D. in clinical psychology and had been working with SCI patients as a clinical psychologist at Ben Taub for several years, at the time this study was conducted.

This was a within-subject design case study with key measures administered before and after each VR DBT® mindfulness training session. Patients received VR DBT® Mindfulness training sessions after they were cleared by the acute surgery team to participate in this study. Patients were also free from infection at the time of the intervention. The measures were administered before and after each VR intervention. During the VR+DBT® skills learning interventions, the participant looked into a pair of wide field of view Oculus Rift DK2 VR goggles (100° field of view diagonal, per eye), and had the illusion of floating down a 3-D computer-generated river (created and owned by bigenvironments.com, see also vrpain.com) while listening to one of three DBT® mindfulness skills training audio tracks. All audio tracks and verbal DBT® treatments used in this study are copyrighted by Marsha Linehan. See below a brief description of each audio track as well as the VR system.

Track 1: Observing Sound (Linehan, 2002; 8.5 min total).

Here is an excerpt “what we are going to do now is practice observing sound….the idea is not to think about sound, the idea is to just notice it…I am going to start by ringing the bell three times….just listen to the bells, how long they last, how they go from loud to soft, just listen, as you focus on your breath coming in and breath coming out. If your mind wanders, bring it back.”

Track 2: Observing visuals: (adapted from Linehan, 2015; 10 min total)

The observing visuals script was adapted to synchronize with images the patient sees in the VR goggles when they are listening to the audio (Navarro-Haro et al., 2016).

Exerpt: “Notice what you see as you float down the river, observe the serenity of the water, attend to what you see in the present moment. If your mind wanders away, just bring it back, gently…to the river.”

Track 3: Wise Mind; (adapted from Linehan, 2002; 8 min long)

The patient listened to these audio instructions while in VR floating down the river in virtual reality. Here is an excerpt “Imagine that you are a small flake of stone, flat, and light. You are now floating slowly and gently through the clear blue water, toward your wise mind…notice what you see, what you feel, as you are floating down toward your wise mind. As you float down the river, observe the serenity of the river, be aware of the calmness, and settle your attention there, within yourself.” “As you reach the center of your self, focus your attention there, in your center, in your wise mind. Come back…to the river..if you find your mind wandering.”

## Virtual reality system

Each participant sat in their hospital bed. After filling out the pre-treatment psychological measures, the patient looked into a pair of Oculus Rift DK2 VR goggles. Patient one had a breathing tube in his neck, and was not allowed to move his head, so the therapist held the VR goggles near his face. Patient 2 wore the VR goggles on his head, and was able to look around in the VR world. Head tracking allowed him to look around the 3D computer virtual world.

**Figure 1:**
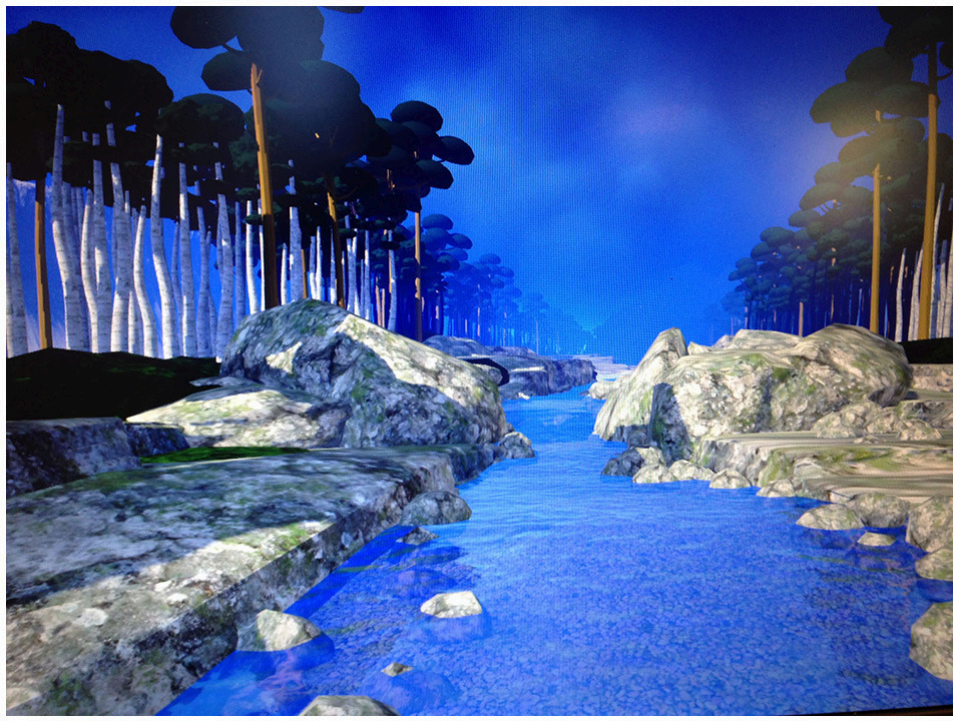
A sample of the virtual reality world the SCI patients experienced in their Oculus Rift DK2 VR goggles while listening to Dr. Linehan's DBT® Mindfulness Skills™ audios (http://behavioraltech.org). Image by www.bigenvironments.com, copyright Hunter Hoffman, www.vrpain.com.

While looking into the virtual reality goggles, each patient looked down river. Near the beginning of the VR session, the patients stayed in one place at the top of the virtual river, as they received verbal DBT® mindfulness skills training exercise instructions from the therapist. Then, the patient began descending slowly down the river while listening to audio instructions directing the patient how to practice mindfulness. Each participant sat in their hospital bed and looked into a pair of head mounted Oculus Rift DK2 VR goggles with 100° diagonal field of view per eye and 960 by 1,080 pixels resolution per eye (75 hz). The goggles were plugged into an MSI GT Series GT72 Dominator Pro G-1252 Gaming Laptop 6th Generation Intel Core i7 6700HQ (2.60 GHz) 16 GB Memory 1 TB HDD 512 GB SSD NVIDIA GeForce GTX 980M 4 GB GDDR5 17.3" Windows 10 Home 64-Bit. The VR world was designed to give the patient the illusion of going inside the 3D computer generated world, where they floated slowly down a computer generated river in VR, with trees, boulders, and mountains. Each patient listened to mindfulness training instructions followed by sounds of birds and crickets chirping, and water sounds, in an immersive computer simulation of floating down a 3-D computer-generated river during the VR DBT® mindfulness intervention (see Figure 1).

Patient 1 listened to Observing and Wise Mind audios while in virtual reality. The patient received “observing visuals” for session 1, “observing sounds” for session 2, and “wise mind” for session 3, and “observing visuals” again, for session 4. While in VR, Patient 2 received “observing visuals,” and “observing sound”. He only had two sessions of VR because his health improved sufficiently that he was discharged before a third session was implemented (i.e., he did not receive the “wise mind” VR DBT® session).

## Assessments

The current study used a measure designed specifically for medical patients. BDI®–FastScreen is a reliable 7-item self-report instrument valuable for measuring the severity of the patient's depression (Beck et al., 2000). The BDI Fast Screen has been shown to have concurrent and discriminative validity (Benedict et al., 2003). The BDI Medical Fast Screen is an abbreviated version of the longer Beck Depression Inventory (Beck et al., 1996a,b). Patients respond to multiple choice questions to measure the severity of the patient's depression. The questions ask patients to rate their hopelessness, irritability, feelings of guilt, etc.

The Spielberger State-Trait Anxiety Inventory (STAI-Y). Internal consistency coefficients range from 0.86 to 0.95, and it has good test-retest reliability coefficients ranging from 0.65 to 0.75 (Spielberger et al., 1983; Spielberger, 1989). The 10 item “STAI-Y short” used in the current study produced scores similar to those obtained with the full form of the STAI-Y (Bergua et al., 2016).

Previous VR DBT® studies used diary based measures of negative and positive emotions (Navarro-Haro et al., 2016; Gomez et al., 2017). The current study introduces new measures using a Graphic Rating Scales question format (Jensen, 2003). The Graphic Rating Scale is a 10-unit horizontal line labeled with number and word descriptors. Patients can easily answer these GRS ratings despite having no previous experience (Tesler et al., 1991). Using single GRS questions, patients rated how “depressed” and “nervous/anxious” and how “emotionally upset” they felt before and after each VR session. Before and after each VR DBT® Mindfulness Skills Training session, each patient also briefly rated on a scale from 0 to 10 the intensity of several primary emotions (sadness, fear, anger, guilt, shame, disgust, and joy, see Ekman, 1992, 2016; Linehan, 2002; Navarro-Haro et al., 2016; Gomez et al., 2017). To measure acute stress disorder/PTSD symptoms, we created a new Graphic Rating Scale of current ASD/PTSD symptoms, adapted from the diagnostic criteria of the DSM-4 (American Psychiatric Association, 2000).

## Results

### Patient 1

Patient 1 tried VR DBT® Mindfulness on four separate occasions (see Table 1). Overall, patient 1 reported feeling less depressed, less nervous/anxious and less emotionally upset, after his VR DBT® sessions. More specifically, as predicted, on Study Day 1, on the 0 to 10 Graphic Rating Scale measures, the patient reported feeling “pretty depressed” (8/10) before the Virtual Reality DBT® Mindfulness skills learning session, which dropped to “moderately depressed” (6/10) after treatment. Patient 1 reported feeling “pretty nervous/anxious” (8/10) before treatment and that dropped to “moderately nervous/anxious” (6/10) after VR DBT® treatment. And patient one's rating of “emotionally upset” dropped from 6/10 to 5/10. Similarly, on items that showed change, the patient showed the predicted reduction in negative primary emotions, after the VR DBT® mindfulness skills training (see Table 1).

**Table 1:** Results.

	**GRS**			**PTSD**					**Emotions**							**Beck inventory pre only**	**STAI**	
	**Depressed**	**Anxious**	**Emotionally upset**	**Thinking**	**Painful memory**	**Felt**	**Upset**	**Avoid**	**Sad**	**Fear**	**Anger**	**Guilt**	**Shame**	**Disgust**	**Joy**		**State**	**Trait**
PRE SESSION 1 S1	8	8	6	0	0	0	0	0	100	75	100	100	100	100	0	17	39	31
Post Session 1 S1	6	6	5	0	0	0	0	0	70	70	90	100	100	100	0		34	
PRE SESSION 2 S1	8	6	6	0	0	0	0	0	70	60	100	100	70	70	100		37	
Post Session 2 S1	5	5	4	0	0	0	0	0	65	50	90	95	70	70	100		33	
PRE SESSION 3 S1	4	4	4	0	0	0	0	0	60	45	50	80	60	60	100		32	
Post Session 3 S1	3	2	2	0	0	0	0	0	50	40	40	80	60	60	100		22	
PRE SESSION 4 S1	5	10	4	0	0	0	0	0	70	50	100	100	100	100	80		34	
Post Session 4 S1	4	9	3	0	0	0	0	0	60	40	80	80	100	100	80		32	
Pre Session 1 S2	6	7	9	9	8	7	0	0	60	60	70	70	100	100	20	6	34	37
Post Session 1 S2	5	6	6	2	0	0	0	2	100	50	90	80	90	100	20		28	
Pre Session 2 S2	3	4	3	2	1	0	0	0	0	0	0	0	0	0	30		28	
Post Session 2 S2	2	3	3	0	0	0	0	0	0	0	0	0	0	0	30		27	

### Patient 2

The patient was injured in a suicide attempt, after he jumped out of a moving vehicle. He was transported to Ben Taub Medical Center, a level 1 trauma center in Houston Texas, where his medical condition was stabilized, but due to damage to the spine including a C7 fracture, the hospitalized patient had bilateral hand numbness and parasthesias, and paresis (partial paralysis of his legs). For a number of psychological issues he was experiencing, he received conventional Cognitive Behavioral Therapy (he did not receive any Standard DBT® therapy sessions). Patient 2 tried VR DBT® Mindfulness on two separate days. As predicted, the SCI patient reported improvements on the psychological measures after the Virtual Reality DBT® Mindfulness intervention. On the GRS ratings (on a zero to 10 rating scale), on Study Day 1, the patient's ratings of feeling depressed dropped from 6 pre-treatment to 5/10 post treatment. Feeling anxious/nervous dropped from 7 pre-treatment to 6 post-treatment, and feeling “emotionally upset” dropped from 9 (severe) pre-treatment to 6 (moderate) post-treatment. On his second session, Patient 2's rating of depressed dropped from 3 before treatment to 2 after treatment, anxious dropped from 4 pre-treatment to 3 post-treatment. The patient rated “emotionally upset” before treatment as 3 and 3 after the VR session (no change).

As shown in Table 1, on Day 1, Patient 2 reported a large reduction in ASD/PTSD symptoms after the Virtual Reality DBT® Mindfulness skills training intervention.

As shown in Table 1, on Study day 1, patient 2's responses on ratings of fear, and shame showed the predicted pattern (lower after VR DBT®). Contrary to predictions, the patient's ratings of sadness, anger and guilt were higher immediately after the VR session. On Day 2, patient 2's ratings of negative emotions were all zeros (or near zero) both pre-and post-treatment. The patient was improving medically, and was discharged after Day 2.

Prior to being enrolled in the current study and trying VR, neither participant had ever attempted to practice mindfulness. According to the patient's comments during the sessions, VR mindfulness DBT® skills training was well accepted by both patients. Both patients were willing to try the technique, and had good experiences. They said VR helped them focus, and helped them practice mindfulness, and both patients saw potential benefits of using VR (including VR DBT® mindfulness skills training) with future spinal injury patients.

## Discussion

The current feasibility study shows encouraging preliminary evidence from the first two SCI patients to try virtual reality enhanced DBT® mindfulness skills training. Both patients accepted the use of VR as part of their treatment. After VR, Patient 1 reported feeling less depressed, less nervous, and less emotionally upset and reported reductions in negative emotions of sadness, fear, anger, guilt, shame, and disgust. Overall, Patient 2 also showed reductions in ratings of depression, nervous/anxious, and reported feeling less “emotionally upset” after VR DBT®, and large reductions in acute stress/PTSD symptoms after his first VR DBT® mindfulness training sessions, but contrary to predictions, patient 2's ratings of sadness, anger, and guilt were higher immediately after the VR session.

The current study has a number of limitations that should be taken into account. Case studies are inherently inconclusive by nature and must be followed up with larger, controlled studies (Campbell and Stanley, 1963). When used in clinical practice, DBT® skills learning typically requires frequent repetition before lasting benefits can be observed. The training in the current study was very limited/infrequent, and long term benefits were not measured. Despite these limitations, the current two case studies are important initial explorations of the feasibility of using Immersive Virtual Reality (VR) enhanced DBT® Mindfulness skills training to help reduce psychological issues of two patients with severe spinal cord injuries. In addition, this is one of the first study on PubMed using the new generation of VR goggles (Oculus Rift VR goggles) with spinal injury patients.

Paralyzed patients lack of mobility limits sensory stimulation. Boredom is a major problem. Virtual Reality allows patients to “go to another place.” There is a growing literature on the use of virtual reality for psychological treatments, e.g., for pain control and treating phobias, PTSD (Hoffman et al., 2000, 2007, 2011, 2014; Difede and Hoffman, 2002; Garcia-Palacios et al., 2002; Difede et al., 2007, 2014; Rizzo et al., 2010). The current study is one of the first to explore the use of VR for psychological treatment of paralyzed or partially paralyzed patients.

Immersive Virtual Reality is becoming widely available to mainstream consumers, and thus has the potential to make Dialectical Behavioral Therapy® Mindfulness skills training available to a much wider number of patient populations, including people with spinal cord injuries. Additional research and development will be needed to determine whether VR DBT® Mindfulness Skills training leads to any long term improvements in outcome.

## Author contributions

All authors listed have made a substantial, direct, and intellectual contribution to the work, and approved it for publication.

### Conflict of interest statement

The authors declare that the research was conducted in the absence of any commercial or financial relationships that could be construed as a potential conflict of interest.
